# Serum KL-6 as a Biomarker of Progression at Any Time in Fibrotic Interstitial Lung Disease

**DOI:** 10.3390/jcm12031173

**Published:** 2023-02-01

**Authors:** Lutz B. Jehn, Ulrich Costabel, Eda Boerner, Julia Wälscher, Dirk Theegarten, Christian Taube, Francesco Bonella

**Affiliations:** 1 Center for Interstitial and Rare Lung Disease, Department of Pneumology, Ruhrlandklinik University Hospital, University of Duisburg-Essen, 45239 Essen, Germany; 2 Institute of Pathology, University Hospital Essen, 45239 Essen, Germany

**Keywords:** progressive ILD, KL-6, biomarker, fibrotic ILD

## Abstract

The development of a progressive phenotype of interstitial lung disease (ILD) is still unpredictable. Whereas tools to predict mortality in ILD exist, scores to predict disease progression are missing. The aim of this study was to investigate whether baseline serum KL-6 as an established marker to assess disease activity in ILD, alone or in combination with clinical variables, could improve stratification of ILD patients according to progression risk at any time. Consecutive patients with fibrotic ILD, followed at our institution between 2008 and 2015, were investigated. Disease progression was defined as relative decline of ≥10% in forced vital capacity (FVC) or ≥15% in diffusing capacity of the lung for carbon monoxide (DLco)% from baseline at any time. Serum KL-6 was measured using an automated immunoassay (Fujirebio Europe, Gent, Belgium). A stepwise logistic regression was performed to select variables to be included in the score. A total of 205 patients (49% idiopathic pulmonary fibrosis (IPF), 51% fibrotic nonspecific interstitial pneumonia (NSIP)) were included, of them 113 (55%) developed disease progression during follow up. Male gender (G) and serum KL-6 strata (K) were significant predictors of progression at regression analysis and were included in the GK score. A threshold of 2 GK score points was best for discriminating patients at high risk versus low risk to develop disease progression at any time. Serum KL-6 concentration, alone or combined in a simple score with gender, allows an effective stratification of ILD patients for risk of disease progression at any time.

## 1. Introduction

Fibrotic interstitial lung disease (ILD) is characterized by dismal outcome and limited treatment options [1,2,3]. Disease progression invariably develops over time in patients with idiopathic pulmonary fibrosis (IPF) and in 18–32% of those with other ILDs [4]. Definition of disease progression in ILD can vary, but usually relies on pulmonary function tests (forced vital capacity (FVC)), worsening of symptoms and/or increase in fibrosis at high-resolution computed tomography (HRCT) scans [5]. The identification of predictors to identify patients at high risk of disease progression, which may require earlier treatment or evaluation for lung transplant, remains a major unmet need.

The GAP (gender, age, physiology) index is a validated prediction tool for mortality risk in idiopathic pulmonary fibrosis (IPF) [6] and other chronic ILDs, such as nonspecific interstitial pneumonia (NSIP) [7]. However, trials to modify the GAP index by weighing the GAP variables, or adding variables such as ethnicity, smoking history, body mass index (BMI), serological markers or even HRCT pattern, resulted in only a slightly better prediction of mortality, especially in IPF and rheumatoid arthritis-associated ILD [8,9]. Importantly, this score has not been validated for predicting disease progression in patients with IPF or other ILDs.

Krebs von den Lungen-6 (KL-6), classified as human MUCIN 1 protein, is mainly produced by regenerating alveolar pneumocytes type II and has been validated as a biomarker of disease activity in ILD [10,11,12]. Data mostly coming from Japanese and Asian studies indicate that elevated KL-6 levels in serum are also associated with the risk of disease progression and mortality in ILD [13], but further studies are needed to validate these promising results in Caucasians.

The aim of our study was to verify whether serum KL-6, alone or combined in a weighted clinical score, could improve stratification of ILD patients for risk of disease progression at any time.

## 2. Materials and Methods

### 2.1. Study Population

Adult patients (≥18 years of age) consecutively diagnosed with IPF and fibrotic NSIP (fNSIP) at our institution between 2008 and 2015 were included in this retrospective analysis. Diagnosis of IPF and fNSIP was revised according to ATS/ERS criteria 2018 and 2013 and had to be confirmed by the institutional ILD-Board [14,15]. None of the patients were taking antifibrotics at the time of blood sampling. Patients were excluded from this study if they had a history of malignancy, or evidence of an active neoplastic process or infection. A total of 40 age-matched healthy subjects without a history or symptoms of lung disease were included to compare serum KL-6 levels. The study was approved by the local Institutional Review Board (IRB) (nr. 06-3170) and all the subjects provided written informed consent.

### 2.2. Pulmonary Function Tests

Measurements of FVC and diffusing capacity of the lung for carbon monoxide (DLco) corrected for hemoglobin (Hb) were performed at the time of ILD diagnosis and KL-6 measurement, using a Jaeger^®^ MasterScreen Body Plethysmograph with SentrySuite^®^ Software (CareFusion, Hoechberg, Germany). Blood hemoglobin concentrations were measured by using the Sysmex XN-550 differential analyser (Sysmex Europe, Norderstedt, Germany). Pulmonary function test results were expressed as percentages of predicted normal values (% pred.) [16].

### 2.3. Definition of Disease Progression

Disease progression was defined as relative decline of ≥10% in FVC or ≥15% in DLco corrected for Hb from baseline at any time. Otherwise, the patients were defined as stable/improved based on the last available follow-up.

### 2.4. GAP Score

GAP score and stage for mortality risk assessment were calculated as previously described [6].

### 2.5. KL-6 Measurement

Blood serum samples were obtained in all subjects at time of ILD diagnosis. The samples were stored at −80 °C until analysis. Serum KL-6 concentrations were measured by Lumipulse^®^ G1200 (Fujirebio Europe, Gent, Belgium), a fully automated chemiluminescent enzyme immunoassay (CLEIA), which is based on a two step-sandwich immunoassay method. Measurements were performed according to the manufacture’s manual by using Lumipulse^®^ G KL-6 Immunoreaction Cartridges (#233207, Fujirebio Europe, Gent, Belgium). Serum KL-6 concentrations were expressed in U/mL. The upper limit of normal (95th percentile) was set at 375 U/mL.

### 2.6. Statistical Analysis

Continuous variables were evaluated for a normal distribution with the Kolmogorov–Smirnov test. Parametric data are presented as mean ± standard deviation (SD) and nonparametric data as medians with interquartile ranges (IQR). Categorical variables are presented as either a percentage of the total, or numerically, as appropriate. Comparisons between the groups were evaluated using a two-tailed *t*-test, Mann–Whitney U or Kruskal–Wallis tests as appropriate for continuous variables, and Chi-squared or Fischer’s exact tests for categorical variables.

Univariable and multivariable Cox’s proportional regression models with hazard ratios were used to identify predictors of disease progression using the clinical variables at baseline (age, gender, BMI, serum KL-6, FVC% pred, DLco% pred and underlying ILD) as explanatory variables, and by stepwise variable selection (backward elimination with a threshold of *p* = 0.05). The selected variables were categorized by using cut-off values determined by receiver operating characteristic (ROC) curve analysis. Multivariable Cox’s proportional regression analysis with the categorized variables was again performed to validate its significance. To sharpen KL-6 level categories, different strata for baseline KL-6 concentrations were tested with Kaplan-Meier analysis and log-rank test. Each selected variable was assigned an integral weight proportional to its odds ratio (OR). The OR was calculated with the exponential of model regression coefficients. The total score was defined as the sum of the values for the selected variables. Subsequently, study subjects were clustered in a high risk (HR) group versus a low risk (LR) group using the optimal threshold determined by a ROC analysis for disease progression at any time. Finally, Kaplan-Meier analysis was used to test the performance of the new stratification score for the risk of disease progression. *p* values of <0.05 were considered statistically significant. All statistical analyses were performed using Addinsoft (2022). XLSTAT statistical and data analysis solution (New York, NY, USA).

## 3. Results

### 3.1. Characteristics of Study Subjects and Baseline Serum KL-6 Levels

We studied 205 subjects with ILD, of them 100 had IPF and 105 fNSIP. In the fNSIP group, 67 patients had idiopathic NSIP, 21 a form associated with systemic autoimmune disease and 17 a form associated with autoimmune features (interstitial pneumonia with autoimmune features, IPAF, details are shown in Table 1). Demographic and laboratory characteristics of the enrolled subjects are shown in Table 1.

**Figure 1:**
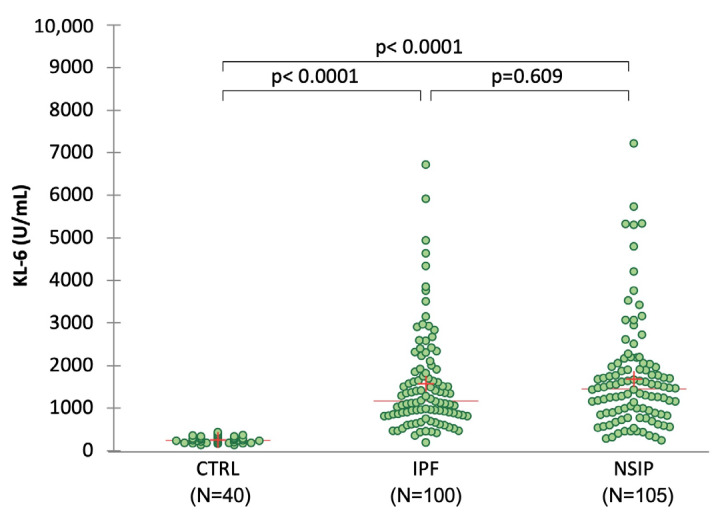
Distribution of baseline serum KL-6 concentrations in the studied subjects. Dots represent single patients. Red line represents the median and red cross the mean values. Significance of the comparisons is shown in the graphic.

The proportion of male patients and smokers was significantly higher in IPF compared to fNSIP (*p* < 0.05 for both comparisons). Patients’ lung function impairment at baseline was similar between IPF and fNSIP. The median baseline KL-6 concentration was 1335 (IQR: 845–1905) U/mL across all patients, and 240 (IQR: 188–296) U/mL in the controls (Figure 1). No significant differences in baseline KL-6 concentrations were observed between IPF (1194 (IQR: 841–1864) U/mL) and fNSIP (1458 (IQR: 883–1905) U/mL), but the median KL-6 levels were significantly higher in ILD patients compared to healthy controls (*p* < 0.0001).

### 3.2. Outcome and Analysis of Predictors of Progression at Any Time

**Figure A1:**
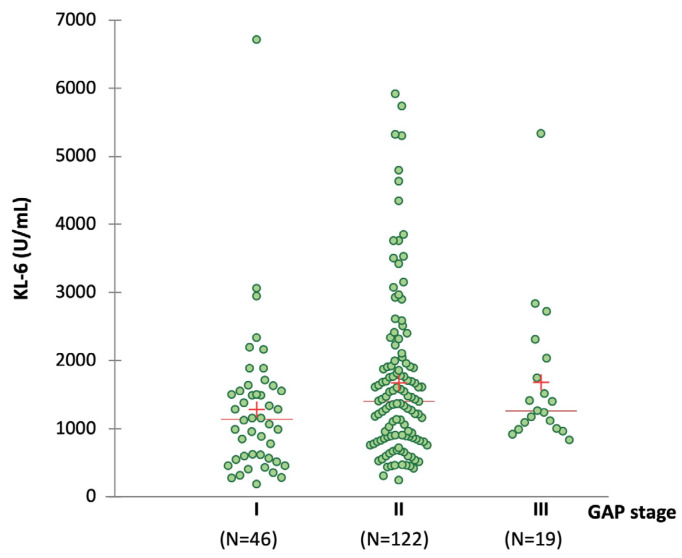
Distribution of baseline serum KL-6 concentrations in the studied subjects according to GAP stage. Dots represent single patients. Red lines represent median and red cross the mean values.

The median follow-up time from initial blood sampling was 33 months (IQR 18–55). A total of 38 patients died or underwent lung transplantation. By using GAP score, 25, 65 and 10% of patients were stratified in stage I, II and III, for risk of mortality, respectively. A trend for an association between the baseline serum KL-6 concentrations and GAP stages was seen (*p* = 0.051; Figure A1).

During a follow-up time of up to 70 months, progression was observed in 113 of 205 patients (55%), of them 61% had IPF and 49% fNSIP, respectively (*p* = 0.09 for IPF versus fNSIP). The mean time to progression was 17.1 ± 13.9 months for all ILD subjects, being significantly shorter in IPF than fNSIP patients (14.6 ± 12.7 versus 18.4 ± 14.0, *p* = 0.045). To identify risk factors of progression at any time, we included age, BMI, KL-6, FVC and DLco as continuous variables, and gender as binary in the regression analysis. We excluded smoking status due to excessive missing data. Table 2 shows the results of the Cox univariate regression analysis for progression at any time. BMI (*p* = 0.022), male gender (*p* = 0.035), underlying ILD (*p* = 0.029) and continuous KL-6 (*p* = 0.017) were significantly associated with disease progression. Since both FVC and DLco at baseline were not associated with progression at any time, we did not perform a regression analysis with GAP index as predictor. When ILD type was included in the analysis, fNSIP was a protective factor for disease progression (*p* = 0.029).

### 3.3. Calculation of KL-6 Strata

**Figure 2:**
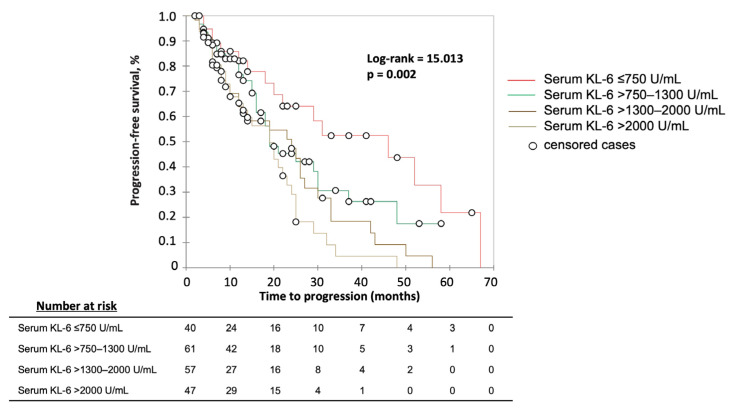
Kaplan–Meier analysis for the risk of disease progression at any time by serum KL-6 levels baseline strata. Significance (Log rank *p*) is shown in the graphic.

We searched for the best strata of serum KL-6 levels correlating with disease progression at any time by using ROC analysis. The threshold of serum KL-6 levels associated with best sensitivity (62%) and specificity (58%) to predict disease progression was found at 1261 U/mL (Area under the ROC curve (AUC) = 0.604, *p* = 0.009). At a targeted sensitivity or specificity of 80%, two other thresholds were found: 752 U/mL (86% sensitivity, 27% specificity) and 1994 U/mL (29% sensitivity, 84% specificity). The performance obtained for the strata of KL-6 ≤750, >750–1300, >1300–2000 U/mL and >2000 U/mL is shown in Figure 2.

### 3.4. Final Score to Stratify Patients for the Risk for Disease Progression

Based on the predictors identified by the first Cox regression analysis, we performed a new Cox regression analysis including gender, BMI, and serum KL-6 strata. Serum KL-6 levels >1300–2000 U/mL (*p* = 0.006), >2000 U/mL (*p* = 0.001) and male gender (*p* = 0.012) were associated with the risk of disease progression at any time (Table 3).

According to these results, the final score for predicting disease progression included male gender and serum KL-6 strata (GK score). Table 4 shows the final GK score with the ORs for disease progression and the points assigned to each variable.

**Figure 3:**
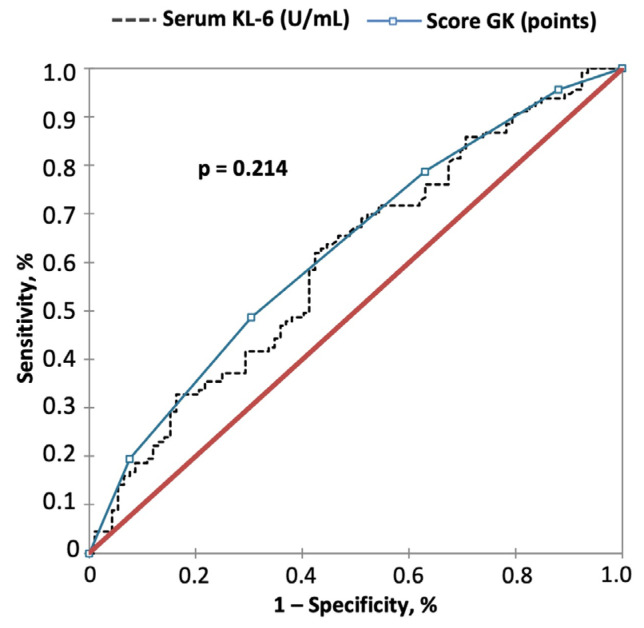
Comparison of GK Score versus KL-6 for prediction of disease progression by using ROC curve analysis. Significance of the comparison is shown in the graphic. PPV, NPV and AUC are indicated in the text of the results.

When included in a Cox regression analysis, GK scoring results were associated with a better performance to predict the risk of disease progression at any time than using the KL-6 strata alone (Table A1). By ROC analysis, the cut-off at 2 GK points yielded a positive predictive value (PPV) of 66% and a negative predictive value (NPV) of 53% (AUC = 0.628, *p* = 0.001) for disease progression at any time, similar to that obtained with serum KL-6 levels alone (*p* = 0.214, Figure 3).

**Figure 4:**
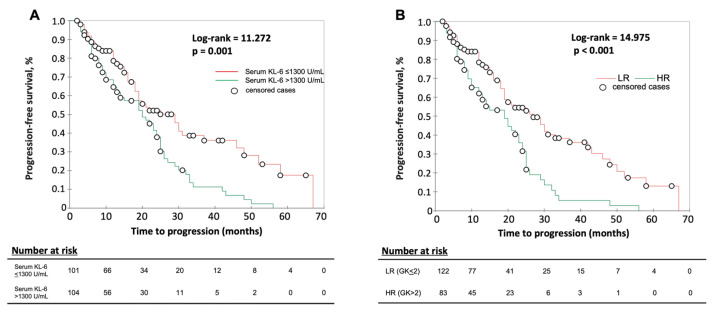
Kaplan–Meier analysis for: (**A**) serum KL-6 at cut-off >1300 U/mL and (**B**) GK score at cut-off >2 points to separate patients at high risk (HR) and at low risk (LR) of disease progression. Higher score values indicate a higher risk for disease progression. Significance (Log rank *p*) is shown in the graphic.

At Kaplan–Meier analysis, the GK score with cut-off >2 points could better separate HR and LR patients than serum KL-6 (>1300 U/mL) alone (Figure 4).

The characteristics of patients according to GK sore risk groups are shown in Table A2.

## 4. Discussion

In our study, we show that serum KL-6 levels alone or included in a simple score with gender, can be effectively used to stratify fibrotic ILD patients for risk of progression at any time. The maximum GK score of four was associated with a hazard ratio of 4.8 for disease progression, which was superior to serum KL-6 strata alone and, therefore, highlights the additional value of gender in the score as an independent risk factor for disease progression in fibrotic ILDs (Table A1).

Over the last two decades, serum KL-6 has been widely investigated as a biomarker for assessing disease severity in ILD, mainly in patients with IPF and connective tissue disease (CTD)-associated ILD [17,18,19,20,21]. An inverse correlation of serum KL-6 levels with impairment of pulmonary function tests, mainly FVC and DLco, has been demonstrated in several cross sectional and longitudinal studies [22,23,24]. One of the major issues in interpreting these results is the heterogeneity of the studied populations in terms of ILD subtype and sample size. A study from Korea found that the semiquantitative grade of fibrosis on HRCT was significantly proportional to the KL-6 serum level, and the optimal cut-off KL-6 value effectively differentiated each fibrosis grade [20]. Whether serum KL-6 can predict functional decline or fibrotic changes at HRCT over time needs further investigation.

In our cohort, half of the patients developed progression over time, IPF patients significantly earlier than those with fNSIP. The analysis of predictors was performed through a multi-step logistic regression. Among the GAP-defining variables, age, gender, FVC and DLco, only male gender was significantly associated with disease progression at any time; hence, we decided not to further investigate the GAP index as a predictor of disease progression. From one side, our findings do not align with previous studies which identified baseline FVC and DLco as independent predictors of disease progression in fibrosing ILD [25,26]. On the other side, despite consistent trends for FVC decline in the IPF population, significant variability in FVC is observed over time, and prior declines, for example, are a poor predictor of future FVC decline [27,28,29]. In consideration of the limitations of our study, especially the treatment heterogeneity and variable disease duration, these findings should be interpreted with caution.

Regression analysis revealed also that underlying ILD was associated with the risk of progression, with fNSIP being protective compared to IPF (Table 2). Since the rate of progression was similar between IPF and fNSIP patients during the follow up time (only the time to progression was different), we decided not to include underlying ILD as a predictor in further analyses.

In the present study, we show that serum KL-6 as a continuous variable or stratified through different cut-offs can be predictive of disease progression at any time. The use of strata allowed us to obtain increasing hazard ratios for the risk of disease progression. The identified cut-offs are consistent with previous findings from our group and other investigators [18,20,30,31,32]. In particular, serum KL-6 levels >1300 U/mL at baseline have been associated with shorter duration before the onset of acute exacerbation in patients with IPF [18]. In a Japanese study on patients with systemic sclerosis-associated ILD, a cut-off of 730 U/mL was found to discriminate active from inactive pulmonary fibrosis with a sensitivity and specificity over 80% [33]. However, we cannot exclude that serum KL-6 levels baseline strata can slightly vary in other study populations due to different ethnicity or heterogeneity of included ILD [34,35].

The inclusion of male gender and serum KL-6 strata in the GK score seems to have the potential to better separate patients at high risk and low risk to develop disease progression at any time if compared to KL-6 alone. Although the performance of the identified GK cut-off two is not satisfactory in terms of positive and negative predictive value (both under 80%), the Kaplan–Meier analysis shows a potential clinical utility for separating patients who will develop disease progression over time from those who do not. The risk of disease progression could depend on different clinical characteristics between the two GK groups. In fact, GK HR patients are characterized by significantly lower DLco values at baseline and higher mortality risk according to the GAP stage distribution compared to LR patients (Table A2). However, we cannot exclude that other factors such as disease duration, treatment type or comorbidities can affect the results of our analysis. Although the present study was underpowered for survival analysis, it can be hypothesized that high GK score patients may also be at higher risk of mortality due to ILD progression [36]. Validation of GK score in a larger multi-center cohort and in further nonfibrotic ILDs is warranted.

Despite the novel findings, our study has several limitations. First, we included only fibrotic ILDs, limiting the validity of our findings for nonfibrotic forms. Second, the median follow-up period in our cohort was about three years, thus limiting the number of the observed progression events and deaths. Third, data on acute exacerbations, a complication known to accelerate disease progression, were not available. Fourth, although no patients received antifibrotics at baseline, for steroids and other immunosuppressive drugs it was not possible to precisely determine the treatment duration and dosage, or whether they were taken alone or in combination. Since immunosuppressive drugs may negatively impact prognosis and disease progression in IPF [37], our findings should be carefully interpreted.

Finally, we did not quantify the extent of fibrosis at HRCT, which might have been included for adjustment of the regression analysis.

## 5. Conclusions

In conclusion, our study shows that baseline serum KL-6 concentration, alone or combined in a simple score with gender, allows an effective stratification of ILD patients for risk of disease progression at any time.

## Figures and Tables

**Table 1:** Demographics and characteristics of the studied subjects.

Variable	Total *n* = 205	IPF *n* = 100	NSIP *n* = 105 *	*p*
Age, y (*n* = 205)	67 (58–73)	68 (61–73.5)	66 (53–72)	0.051
Male gender, *n* (%) (*n* = 205)	132 (64)	79 (79)	53 (50.5)	<0.0001
BMI, kg/m^2^, (*n* = 205)	28 (25–31)	27 (25–31)	28 (26–31)	0.341
Smoking history (yes/no), (*n* = 107)	69/38	42/5	41/19	0.013
FVC, % pred, (*n* = 205)	69 (56–76)	69 (57–75.5)	67 (55–76)	0.763
DLco, % pred, (*n* = 187)	46 (37–57)	46 (37.5–57)	46 (37–57)	0.938
GAP stages, I/II/III (%), (*n* = 187)	25/65/10	21/68/11	28/63/9	0.511
KL-6, U/mL, (*n* = 205)	1335 (845–1905)	1194 (841–1864)	1458 (883–1905)	0.290

If not otherwise indicated, values are expressed as median (IQR). * 67 patients had idiopathic NSIP, 21 had NSIP associated with systemic autoimmune disease (10 RA-ILD, 2 inflammatory myositis, 6 SSc-ILD, 1 Temporal arteritis, 2 Sjogren Syndrome) and 17 had NSIP associated with autoimmune features (IPAF). Abbreviations: BMI = body mass index; FVC = forced vital capacity; DLco = diffusing capacity of the lung for carbon monoxide; GAP = gender, age, physiology; IPAF: interstitial pneumonia with autoimmune features; IPF: idiopathic pulmonary fibrosis; IQR = interquartile range; *n* = number; KL-6 = Krebs von den Lungen-6; NSIP = nonspecific interstitial pneumonia; RA: rheumatoid arthritis; SSc: systemic sclerosis; Y = years.

**Table 2:** Cox regression analysis for predictors of lung function decline at any time.

Variable	Wald Chi-Square	HR	HR 95% CI	OR *	*p* **
Age, y	1.132	1.010	0.991–1.030	1.024	0.287
Gender (ref. M)	4.454	1.660	1.037–2.657	3.210	0.035
BMI, kg/m^2^	5.279	1.050	1.007–1.095	1.119	0.022
KL-6 (U/mL)	5.687	1.000	1.000–1.000	1.000	0.017
FVC, % pred	0.321	0.995	0.979–1.012	0.989	0.571
DLco, % pred	0.168	0.997	0.982–1.012	0.993	0.682
Underlying ILD (ref. NSIP)	4.739	0.616	0.000–0.953	0.328	0.029

* ORs were calculated as exponential of regression coefficient values derived from logistic regression (event = progression) and are shown for completeness. ** refers to HR calculation through Cox regression analysis. Abbreviations: BMI = body mass index; FVC = forced vital capacity; DLco = diffusing capacity of the lung for carbon monoxide; GAP = gender, age, physiology; HR = hazard ratio, ILD = interstitial lung disease; IQR = interquartile range; *n* = number; KL-6 = Krebs von den Lungen-6; NSIP = nonspecific interstitial pneumonia; OR = odds ratio; y = years.

**Table 3:** Cox regression model for predictors of lung function decline at any time.

Variable	Wald Chi-Square	HR	HR 95% CI	OR *	*p* **
Gender (ref. M)	6.329	1.689	1.123–2.541	3.343	0.012
BMI (ref. >28 kg/m^2^)	0.760	1.187	0.808–1.743	1.483	0.383
Serum KL-6 (ref. >750–1300 U/mL)	2.521	1.670	0.887–3.146	3.258	0.112
Serum KL-6 (ref. >1300–2000 U/mL)	7.600	2.413	1.290–4.513	7.600	0.006
Serum KL-6 (ref. >2000 U/mL)	10.648	2.924	1.535–5.571	11.832	0.001

All serum KL-6 strata were compared to stratum ≤750 U/mL. * ORs were calculated as exponential of regression coefficient values derived from logistic regression (event = progression) and are shown for completeness. ** refers to HR calculation through Cox regression analysis. Abbreviations: BMI = body mass index; HR = hazard ratio; KL-6 = Krebs von den Lungen-6; M = male; OR = odds ratio.

**Table 4:** GK score based on gender and serum KL-6 levels at baseline to predict disease progression at any time.

Variable	*p*	OR *	Points
Gender (ref. M)	0.014	3.209	1
KL-6 > 750–1300 U/mL	0.091	3.484	1
KL-6 > 1300–2000 U/mL	0.005	7.692	2
KL-6 > 2000 U/ml	0.001	13.042	3

To the stratum KL-6 ≤ 750 U/mL 0 points were assigned. * ORs were calculated as exponential of regression coefficient values. Abbreviations: M = male; KL-6 = Krebs von den Lungen-6; OR = odds ratio.

## Data Availability

All data generated or analyzed during this study are included in this article and Appendix A and Appendix B. Further enquiries can be directed to the corresponding author.

## Study Approval Statement

This study protocol was reviewed and approved by the local Institutional Review Board (Ethik-Kommission der Medizinischen Fakultät der Universität Duisburg-Essen), approval number (06-3170, date of approval: 3 June 2008).

## Appendix A

**Table A1:** Comparison of statistical performance of baseline serum KL-6 strata alone versus GK scores as predictors of disease progression at any time.

Variable	Wald Chi-Square	HR *	HR 95% CI	*p*
Serum KL-6 (ref. > 750–1300 U/mL)	2.521	1.670	0.887–3.146	0.112
Serum KL-6 (ref. > 1300–2000 U/mL)	7.600	2.413	1.290–4.513	0.006
Serum KL-6 (ref. > 2000 U/mL)	10.648	2.924	1.535–5.571	0.001
GK = 1	0.938	1.633	0.605–4.404	0.333
GK = 2	4.601	2.814	1.093–7.242	0.032
GK = 3	8.109	3.972	1.537–10.265	0.004
GK = 4	9.788	4.829	1.801–12.952	0.002

All serum KL-6 strata were compared to stratum ≤750 U/mL. * HRs were obtained by Cox regression. Abbreviations: G = gender; HR = hazard ratio; K = KL-6; KL-6 = Krebs von den Lungen-6.

**Table A2:** Baseline characteristics of patients according to GK risk scores for disease progression.

Variable	Total *	HR by GK Scoring **	LR by GK Scoring	*p*
	*n* = 205	*n* = 83	*n* = 122	
Age, Y (IQR)	67 (58–67)	66 (57.5–72)	68 (59–73.5)	0.304
BMI, kg/m^2^ (IQR)	28 (25–31)	28.1 (26–32)	27.5 (25–31)	0.172
Male gender, *n* (%)	132 (64)	65 (78)	67 (55)	0.0001
ILD (IPF/fNSIP), *n* (%)	100 (49)/105 (51)	44 (53)/39 (47)	56 (46)/66 (54)	0.389
FVC, % pred (IQR)	69 (56–76)	66 (54.5–73)	70 (58–79)	0.050
DLco, % pred (IQR) *	46 (37–57)	42 (35.5–49)	51 (40–61)	0.0004
GAP stages, I/II/III (%)	25/65/10	13/76/11	32/58/10	0.005/0.018/0.809
KL6, U/mL (IQR)	1335 (845–1905)	2191 (1604–3065)	911 (615–1214)	<0.0001

If not otherwise indicated, values are expressed as median (IQR). Mann–Whitney test was used for quantitative variables, Fisher’s exact test was used for qualitative variables. * 18 DLco missing. ** GK scoring > 2 points. Abbreviations: BMI = body mass index; FVC = forced vital capacity; DLco = diffusing capacity of the lung for carbon monoxide; GAP = gender, age, physiology; HR = high risk; IQR = interquartile range; LR = low risk; *n* = number; KL-6 = Krebs von den Lungen-6; y = years.
